# New Editions of Standard Works

**Published:** 1840-01-01

**Authors:** 


### NEW EDITIONS OF STANDARD WORKS.

I. PRACTICAL SURGERY; with One Hundred and Thirty Engravings on Wood. By *Robert Liston*. Second Edition.

MR. LISTON informs us:—

" A Second Edition having been already called for, the letter-press has been revised and corrected with care. Various alterations and additions have been made, and a few new wood-cuts added; some have also been engraved anew, in place of such as did not exhibit the subjects distinctly. These improvements, it is hoped, may render the work both more useful to surgical pupils, and more deserving the patronage of the profession at large."

The short time which has elapsed since the publication of the first edition of this work, is sufficient evidence of the estimation in which it is held. It is the first original treatise on operative surgery, at least of any importance, which has been brought out in this country. Our other manuals have been avowedly or really taken from the French, and differ in many respects from the present. This gives us the results of Mr. Liston's experience, which may be safely admitted to have been considerable, and to possess strong claims on our attention.

It would be absurd to attempt an analysis of a book, which is in a great degree elementary, and which is in the hands of so many surgeons. We cannot, however, resist extracting, for the sake of those who have it not, some of the valuable suggestions which abound in it. Of course, we must dispense with any thing like connected order in our quotations.

*Necessity of studying the Art of Operating.*

We agree with Mr. Liston that it is the bounden duty of every one engaging in the practice of our profession, in addition to a correct knowledge of the *science of surgery*, the causes, symptoms, and consequences of disease, the signs of injuries, the morbid changes in the tissues involved, to make, moreover, the *art of operating* the subject of most careful and diligent study. And probably he is not far wrong when he observes:—

The art of operating has, even by many of those in prominent situations, been too lightly considered, and too little practised on the dead body. The foundation of the study of the art of operating must be laid in the dissecting-room, and it is only when we have acquired dexterity on the dead subject, that we can be justified in interfering with the living. Many poor creatures have been sacrificed in consequence of the ignorance, carelessness, and self-sufficiency even of scientific professors, who have either despised or neglected the study of surgical anatomy, the considerations of what may arise during this or the other operation, and the due education of their fingers. The infliction of unnecessary pain, through want of adroitness in the use of instruments, by protraction of any proceeding, the hazarding in the slightest degree the safety of any one who puts confidence in us, who trusts us with his life, or of one who, as in public practice, is, as it were, by chance, and without the means of appeal, thrown into our hands, cannot by any means be palliated or defended—it is, in point of fact, highly criminal.

*Dressing of Wounds.*

"A sort of routine practice has been long pursued in dressing wounds. They are put together without delay, and their edges squeezed into apposition and retained so by various means, such as sutures, plasters, compresses, and bandages. They are carefully covered up and concealed from our view for a certain number of days. Then the envelopes of cotton and flannel, the compress cloths, the pledgets of healing ointment, and plasters are taken away, loaded with putrid exhalations, and a profusion of bloody, ill-digested, fœtid matter. A basin is forthwith held under the injured part, and the exposed and tender surface is deluged with water from a sponge, and then well squeezed and wiped. Then comes a re-application of retentive bandage, of the plaster, of the grease mixed with drying powder, all surmounted by some absorbent stuff, as charpie or tow, to soak up the discharge. This is not unaccompanied by pain, often more complained of than that attendant upon the original injury or operation. This process is repeated day after day, the patient is kept in a state of constant excitement, and often, worn out by suffering, discharge and hectic fever, falls a victim to the practice. It would here serve no purpose to detail the mode of cutting the plasters, and of spreading the pledgets; nor would there be any use in giving an opinion as to whether a mixture of the earth called armenian bole, or the impure oxyde of zinc, the tutty or calamine, was the best ingredient to put into the digestive, drying, healing unguents, or as to whether they should be compounded of one kind of animal fat and vegetable oil, or another; nor would any good come of stating how the soiled bandages and filthy straps are to be removed, whether they are to be cut off or removed first from one end and then from the other. The system is a bad one, the applications filthy and abominable; the whole proceeding outrages nature and common sense. The wound is, as it were, put in a forcing-bed; excited action, beyond what is required, is hurried on, and the consequence is, that union seldom, if ever, can or does take place. A suppurating surface, on the contrary, with profuse discharge, and a very tedious cure, if any, is obtained."

This is strong language, but perhaps it is not altogether unwarrantable. There can be no doubt of the existence, indeed the prevalence of the evil that Mr. Liston reprobates; but, perhaps, he carries his horror of all dressings save water rather far. With the utmost love for the pure element, we are disposed to hesitate before we subscribe to the oath of the surgical tea-totallers.

*Mr. Liston's method of dressing.*—Surfaces are not disposed to unite for many hours after the division and separation have occurred. So long as oozing continues, there is no good end to be achieved by their close apposition. It is only when reaction has occurred, when the vascular excitement around the solution of continuity has taken place, and the circulation has been roused, when plastic matter begins to be secreted and thrown out, that the process can be expected to commence. The edges of a large wound, as that resulting from amputation of the extremities, may be approximated in part so soon as the bleeding from the principal vessels and larger branches has been arrested. But the close apposition, and the application of all the retentive means, had better be delayed for six or eight hours at least. In the interval, extreme sensibility of the injured parts may be abated, the oozing moderated, and the chance of secondary hæmorrhage much diminished, by covering the parts with lint dipped in cold water, and frequently renewed; or, as after any of the greater amputations, for instance, a piece of lint may be placed betwixt the flaps, and a constant irrigation of the exposed surface kept up for some time by threads passing from a vessel containing cold water. This practice is the best that can be resorted to also in accidental wounds, those of the incised kind, that are fresh, and bleed freely. In these last, after hæmorrhage has entirely ceased, applications of an agreeable warmth should be substituted, such as poultice of bread and water, or, what is much to be preferred for its simplicity, lint of thick texture, and of sufficient size to cover the wound, soaked in tepid water, and that overlaid with an ample piece of oiled silk, to prevent evaporation. Heat and moisture, by which qualities a poultice produces its soothing and beneficial effects, by which the surface is relaxed, its capillary circulation encouraged, and discharge promoted, are thus amply afforded, without any of the weight, putrefactive fermentation, stench and filth, which is inseparable even from the best and most scientifically contrived epithems and cataplasms; of course the approximating such wounds is effected, as much as possible, by position, and by the same means the return of blood is favoured, and engorgement of the vessels and inflammatory swelling prevented in a very great degree.

He goes on to observe:—

"As the solution of continuity is filled up, the edges are approximated, and during the cicatrisation the same means must be persevered in, in order to promote the process. Support may be given in some stages of the cure by bandage, and occasionally by plasters; care must, however, be taken that this is not partial. It is seldom, indeed, that any good is seen to arise out of the drawing together by plasters of the edges of a recent solution of continuity, during the process of granulation and cicatrisation. The contraction of the part takes place naturally, and generally with sufficient rapidity. It is often retarded by the means which are foolishly employed to hurry it on. The granulations are absorbed, the surface of the sore becomes foul, the discharge thin and offensive, and if the plan is persevered in, inflammation of the surrounding skin will follow, with extension of the sore by ulcerative absorption. There is a period, however, at which the edges of a solution of continuity in a state of suppuration may sometimes be approximated and retained with every prospect of abridging the cure, provided the surrounding parts are in a sufficiently lax state; viz. when the discharge begins to thicken and diminish in quantity, when the granulations are florid, small, and acuminated; then, indeed, rapid adhesion often takes place."

We must say that we think Mr. Liston rather underrates the effects of judicious strapping, as well as of stimulating applications. Of course both the one and the other may be abused as well as mismanaged. But, properly employed, they do appear to be not altogether useless.

Our author proceeds:—

"When the coverings have been smoothly and cleverly cut, and in the direction of the fibres, when there is an abundance of these as regards the deeper parts, then, after the surface has become glazed, (the vessels having been tied, and the cold water assiduously applied for some hours,) the surrounding skin previously shaved, is made thoroughly dry; coagula are removed, the edges are put carefully and neatly into contact, and retained by narrow slips of adhesive plaster placed at intervals; that commonly used is not well suited for the purpose; it does not retain its hold sufficiently long; it is loosened by discharge, it heats the surface and often gives rise to erythema. For many years a better sort of plaster has been used very successfully by me and some of my colleagues in the Royal Infirmary of Edinburgh, and in the North London Hospital, as well as in private practice. It is composed of a solution of isinglass in spirit, and may be spread for use, as occasion requires, on slips of oiled silk; on silk glazed on one side only, and on the unglazed side. But this plaster can now be had, ready for use, admirably prepared by Messrs. Fisher and Toller, Conduit Street. It is cut into strips of the desired breadth, and the adhesive matter dissolved immediately before it is employed by the application of a hot moist sponge. This composition becomes sufficiently adherent; it keeps its hold often to the end of the cure, and it is quite unirritating. Being transparent, the plaster does not prevent any untoward process that may be going on underneath from being observed, and if any fluid collects, an opening can be snipped for its escape. If, as may be often deemed necessary, a few points of suture have been made, these can be removed by cutting the thread shortly after the fixing of the plasters, and within twenty-four hours. No other dressing need be employed in the first instance—no compress, no pledget, no bandage. These applications may give rise to unpleasant effects, they are not wanted, and do no good; they in no way promote the object in view, viz. the union of the wound, and the cure of the patient at the end of eight or ten days. A roller may be applied in a few days after some amputations, in order to encourage the subsidence of any slight œdema that may have arisen, and to bring the stump into a nice form. At an earlier period, the use of any dresssing whatever is productive of pain; it heats the part, and encourages discharge of blood and formation of matter. The discharge that does take place in the light and simple mode of managing wounds here recommended, is wiped from the surrounding skin, as it flows out, and from the taffeta or glazed cloth on which the part lies."

The isinglass plaister, to which Mr. Liston alludes, is certainly a very excellent application. Its introduction into use must be considered an improvement.

*Division of the Lateral Ligament for Dislocation of a Phalanx.*

Mr. Liston observes :—

The reduction of luxation of the proximal phalanx of the thumb, on the posterior aspect of the metacarpal bones, has occasionally been found puzzling enough, and it is never accomplished without some little trouble. This difficulty has been attributed to the action of the short flexor, and to the tight embrace of the lateral ligament round the neck of the bone. By making the extension across the palm of the hand, the one obstacle is done away with; but the strength and rigidity of the ligaments are not in general easily overcome. Before the reduction has been effected, it has been found necessary to divide one of the ligaments; the external is most easily reached and cut across by the introduction of a narrow-bladed knife (such a one as is used by some practitioners of surgery for exploring tumors) through the skin at some distance, and by directing its edge against the resisting part.

In the "Elements of Surgery" I have related one case of a very old man, much intoxicated, in which it was found absolutely necessary to perform this operation before the reduction could be accomplished; and shortly before giving up the charge of the Edinburgh Hospital, a lad, under fifteen years of age, was admitted with luxation of this joint of fourteen days' duration. In his case it was impossible to effect the replacement until the ligament had been divided. He returned within a week, having again fallen and displaced the bones. The reduction was on the second occasion made readily enough.

Our author does not appear to be aware of the influence of the glenoid ligament in preventing the reduction of the dislocation of the distal end of the metacarpal bone or phalanx on the palmar surface of the proximal end of the phalanx in connection with it. That operates more powerfully, from its anatomical construction, than the lateral ligaments, indeed the latter being partially implanted into the former they co-operate in occasioning the difficulty. The division of the lateral ligament cannot be altogether free from hazard, and perhaps it would be better to avoid it in persons of an irritable, or of otherwise a bad habit of body.

*The Gum Splint for Diseased Joints.*

A variety of methods have lately been resorted to for insuring the quietude of diseased joints. Every body knows the old paste-board splint—now we have the undressed leather splint—and the white of egg splint—and the gum splint —and a dozen more. The following is Mr. Liston's description of a gum splint.

A splint, similar to that used by the old surgeons, composed of some soft substance, as tow and albumen, described by Scultetus, (and delineated by him even to the eggs on a platter,) is applied; we use coarse soft lint soaked in a strong solution of gum acacia; it is laid on in strips over the side and pelvis, from the short ribs to the knee, and made to embrace the limb fully. A layer of dry lint is first applied, and then two or three others, soaked in the mucilage, follow; this is covered by a fold or two of coarse calico, and the whole is retained by a roller. In cases where the limb has been retained for a long period adducted and bent, and where some little trouble and uneasiness have arisen in placing it in a favourable position, it will be advisable to preserve it so by the use of the thigh-splint, as for fracture, during some twenty or twenty-four hours, until the composition dries, and the splint has adapted itself closely to the parts.

The gum splint can be made of any form, so as to allow of its being taken off, trimmed, and lined with wash-leather, or protected with a layer of oil-silk and reapplied with a clean bandage. It can also be fashioned so as to leave any part exposed, in order to admit of dressings; the discharge from issues and abscesses can thus be allowed to escape, and the parts attended to without disturbing the limb. Two splints can be formed instead of one; in fact, the apparatus can be varied as circumstances demand, and it is applicable in a great variety of cases, and to any articulation. A perfect mould is formed of the part, and this can be applied with any degree of tightness that is suitable and comfortable to the patient.

*Removal of the Sterno-mastoid Muscle.*

"I had once occasion to remove the sterno-mastoid muscle of one side, involved in a sarcomatous tumour from its origin to its insertion; the most fastidious critic will not refuse the term sarcoma to this morbid growth, though, in all probability, the muscular fibres may have been involved secondarily. The tumour was limited by a cellular sheath, yet the dissection was difficult and extensive. The patient made a good recovery, and no mal-position of the head followed. The trapezius, probably, of the opposite side had done the duty of the diseased muscle for a considerable time before its removal."

*Ligature of the Carotid, prior to Operations on the Jaws, &c. unnecessary.*

"The operation has been resorted to, in order to arrest the growth of tumours of the face and jaws, for which purpose it must be ineffectual; and it has also been performed as a preliminary step to the removal of such diseases. The suffering of the patient is thus much prolonged, without his safety being at all enhanced, or the dissection of the tumour in any way facilitated. The flow of blood is quite as effectually commanded by pressure with the fingers on the common carotid of the affected side; pressure on both at the same time has given rise to the most alarming convulsive movements in some cases, after great loss of blood had occurred. Even pressure on one trunk is not demanded in many of the operations in question. I have, I believe, had fully as much experience in the management of tumours of the mouth and jaws, and of the face and neck, as any surgeon in this or any other country, and have never seen occasion to tie the carotid previous to or during the operations for their removal. I have never regretted omitting this supposed precautionary measure."

*Is temporal arteriotomy preferable to cupping?* "The propriety of taking away blood from this vessel in particular cases, in preference to abstracting it from the general mass of circulating fluid by opening a vein, cannot be discussed here; as a mode of local bleeding, it is certainly to be preferred to the scarification and cupping of the temples. This last is more painful, and produces deformity; the same troublesome consequences, besides, result occasionally as from arteriotomy. It is not long since I assisted my colleague, Mr. S. Cooper, in operating on a case of aneurysm of the anterior branch of the temporal, consequent upon the application of the cupping-scarificator."

We do not feel quite disposed to go with Mr. Liston here. So far as we have seen, if bad consequences follow cupping once, they succeed arteriotomy twice or thrice. Cupping is, we think, the *least* painful. Cupping entails no pressure or fall afterwards. And cupping, after all, in many cases, is the most effectual.

*A Case of Spontaneous Aneurysm of the Brachial, at the Bend of the Arm.*— "Disease of the coats of the arteries of the upper extremity, to a great extent, is not common, and very few cases of true aneurysm lower than the axilla are met with or mentioned in surgical works. The surgeon will accordingly seldom be called upon to perform any operation for spontaneous aneurysm (aneurysm from disease of the coats) at the bend of the arm. I have treated but one such case in the person of an old ship's carpenter. Whilst at work as usual, he felt something snap in his arm, a pulsating tumour was soon after noticed, and before I was asked to see him, by Mr. Cheyne of Leith, it had attained, during four months, fully the size of a hen's egg, and was evidently in part made up of solid matter. The brachial was tied, and everything went on favourably."

*Aneurysm of the Radial Artery in the Palm, cured by Ligatnre of the Radial and the Ulnar.*—This case was communicated to Mr. Liston by Mr. Pilcher, the operator.

"A working goldsmith, about forty years of age, of a gouty diathesis, had a tumour formed beneath the ball of the right thumb; it was mistaken for an abscess; on careful examination, I discovered it to be an aneurysm, and believed it to be situated between the abductor pollicis and abductor indicis, and to be a disease of the radial artery at its terminal division, probably induced by the repeated, though slight, blows from the handle of the hammer, which his occupation constantly obliged him to use. I proposed to tie the radial and ulnar arteries immediately above the wrist, provided the ligature around the radial was ineffective in diminishing the tumour and arresting its pulsation. My expectations were realised; the closure of the radial artery was attended with diminution in the size and pulsation of the tumour, but still both remained to rather less than half the previous degree. I immediately tied the ulnar, when the tumour was much reduced in size, and the pulsation completely: slight secondary hæmorrhage occurred from one of the arteries at the seat of the ligature two or three days after the operation, which was arrested by cold water; the case progressed without any further untoward symptom, and was attended with a perfect cure."

*New Noses.*—Mr. Liston speaks very warmly in favour of this branch of art. There can be no doubt of the advantage of a good nose. It is almost, of itself, a passport to society. As the song says,

"Although 'tis confess'd that the prejudice goes
Very much in the favour of wearing a nose,
Yet a nose shouldn't look like a snout."

Much less should a nose be wanting altogether. Mr. Liston protests :—

"Were those who seem to despise the repairing of such deformity by surgical interference, holding it as a frivolity and little better than operative quackery, to witness the utter wretchedness of the mutilated object, and how eagerly he would cling to any hope, however slender, of bettering his condition, prejudice against rhino-plastics ought rapidly to thaw before the warmth of aroused feelings of humanity. If not, put the scoffer in the place of the patient,—let him lose his nose, reputably or otherwise, allow him some weeks of consequent misery and degradation, then bring him in contact with a skilful and dexterous surgeon, who despises no part of his professional practice that is to benefit his fellow-creatures, and thinks it not beneath him to make noses as well as stumps, and there can be little doubt that ere long the rhino-plastic art will have obtained an addition to the list of its supporters, of a sure and zealous proselyte."

Certainly the Chapter on new noses, (which by the bye beats Sterne on noses all to sticks) is well adapted to serve the nasal cause, for it gives an excellent account of the history and method of making noses, Roman, Greek, or Pug. Mr. Liston, indeed, is modest. He disclaims classical noses.

"If properly constructed, though not in accordance with either the Roman or Grecian contour, as their predecessors may have been, still they are noses to do well in the world. They are literally very passable noses; for many have I known to pass those of my own making, without remarking anything particular in the features of the wearers. And surely none can deny that the worst made nose is infinitely superior to the horrid cavern which previously struck the passer-by with pity and disgust. As a fair sample of the manufacture, I annex representations of my first case, before and after operation."

For the mode of carving the nose we must refer to the work itself, which affords very ample and very satisfactory information. We extract only the concluding observations :—

In all these operations on the face, there is no inconsiderable risk of erysipelas ; and the surgeon must not only be on his guard against this untoward occurrence, but be prepared to treat it actively. To prevent it, as well as to promote adhesion, the wounds, as has already been stated, are brought together simply by suture, all heating and heavy dressings are avoided, and the sutures are cut out as soon as they can possibly be dispensed with. Notwithstanding these precautions, and attention to the state of the system, particularly in securing healthy action in the primæ viæ, should erysipelas occur, besides the usual constitutional treatment, blood must be taken freely from the transplanted part, and its neighbourhood, by punctures. If the attack be mild, it will yield to this treatment ; but if infiltration be going on, or even threatened, recourse must be had at once to free incision, not only in the neighbourhood of the transplanted part, but in that part itself. At this the surgeon must not hesitate ; for there is no risk, under the circumstances, of the vitality being lost in consequence of even very free incision ; and it is now well known that wounds in the transplanted parts heal with more rapidity than in the original formations. The incisions should be made at an early period of the accession, it being of the utmost importance to cut the disease short; for erysipelas, so induced, not only interferes with the process of adhesion, and endangers either partial or total sloughing of the part, but, as experience has shown, may terminate fatally.

*Mode of removing Encysted Tumors.*—No dissection is required for the removal of these encysted tumors of the scalp, unless indeed they have obtained an enormous size, and, from long residence and other causes, adhere firmly. In general, when the swelling is about the size of a filbert compressed, or even the bulk of a chestnut, it can be taken out, and lifted, as it were, by the forceps from its bed of fine cellular tissue upon merely dividing the super-imposed scalp. The least painful and most expeditious mode is to transfix the tumor with a narrow bladed knife in the direction of the fibres, with the back of the instrument directed towards, the cranium, and to divide thus at once the cyst and skin by cutting outwards, a little of the hair having been previously clipped off the swelling. The edges of the incision are now slightly separated with the forefinger and thumb of the left hand, and the common dissecting forceps (an instrument seldom well made) used for seizing the edge of the cyst and extracting it. The few drops of blood that escaped are wiped up, and no dressing whatever need be applied; some five or six tumors may thus be removed, without much pain to the patient, at one sitting. Sometimes a small clot fills the cavity, and may require being squeezed out a few days after the operation. When the tumor is very small, a slightly curved sharp-pointed knife, the edge on the concave aspect, will be found to answer best. Unless the tumor has attained an enormous size, and has adhered firmly, no portion of skin should be removed.

Encysted tumors about the forehead and eyebrows are seldom permitted to attain any large size, but their adhesions are generally firm, and prove troublesome; dissection is sometimes required in their removal. The difficulty will be much obviated by making the first incision sufficiently free. The cyst is thus fully exposed, and may be removed unopened. These tumors contain curdy or sebaceous matter, with hairs. These are loose, or are seen springing from the inner surface of the bag. The extirpation of such tumors must be clean and perfect, otherwise the secretion is continued, and, as a consequence, there is reproduction of the disease, or else a fistulous opening continues furnishing thin gleety discharge, and only got rid of by incision and removal of the remnant of the secreting surface.

*Encysted and Solid Tumors of the Eyelids.*—Mr. Liston's account of these is not bad:—Solid and encysted tumors are frequently met with about the eyelids; the former generally grow from the conjunctival surface, the latter are situated betwixt the two layers, and often seen to be developed in the substance of the cartilage. The solid tumor may be fully exposed by everting the eyelid; it is seized with a fine hook or forceps and dissected off, along with the portion of mucous membrane from which it proceeds. Some care will be required in removing such tumors growing from the inner corner, (encanthis,) if they have attained considerable size, in order to avoid interference with any part of the apparatus for conveying away the secretions. The cysts in the eyelids generally contain a glairy fluid, they are thin and very adherent, they attain a considerable size, as regards the part in which they are situated, and occasion deformity and inconvenience; the skin is projected and often discoloured; sometimes more than one exists in the same eyelid. They occur equally in the lower and upper lid, perhaps most frequently in the latter. There is no possibility of dissecting out the cyst entire, and, in attempts to do so, a perforation has been made through both skin and conjunctiva without the object having been accomplished. The correct and effectual procedure is, after everting the eyelid, (and this can be done perfectly well, after some little practice, as with the view of attacking granulations on the surface of the upper lid, or removing foreign bodies, by a dexterous application of the fingers, and without the aid of a probe or other instrument,) and exposing the little projection within, to open this pretty freely with the point of a fine knife, a crucial incision may be made if the cyst be large, the contents escape, and the bleeding is allowed to cease. The cavity is completely cleared, and the sac disturbed and lacerated by turning about a sharp-pointed probe in its cavity. If a thorough and radical cure is desired, it will be advisable to take more effectual means for the obliteration of the cavity, and for this purpose a small pointed piece of the potassa fusa may be introduced into it for an instant. This must be done with great caution, the melting caustic being washed away with a bit of lint dipped in vinegar and water, and the bulb of the eye afterwards protected for a short time by the interposition of a small slip of lint, or old linen, smeared with oil, or some mild liniment or ointment. The safest and best mode of applying the caustic is with a very small hollow ball probe.

*Plugging the Posterior Nares.*—This is easily effected by the introduction of a ligature from the mouth forwards into the nostril: a loop of silver or other wire will answer as well as any of the complicated instruments sold for the purpose of conveying the ligature. This is passed along the floor of the nostril from before backwards; it is seen and laid hold of by the fingers, or blunt hook, or forceps, in the pharynx, and brought forward; to this is attached a strong thread ligature, which is drawn into the passage by the removal of the wire. To the middle of the ligature, a piece of lint, the bulk of the first joint of the thumb, larger or smaller, according to the size and sex of the patient, is attached in a noose. This is conducted by the finger behind the velum, and pulled firmly into the cavity. The anterior opening is then plugged with lint also. The dossil is easily removed in a day or two, after washing out the coagula, by pulling at the string and assisting in its dislodgement, by the introduction, from before, of a strong blunt probe. It is seldom necessary to adopt this proceeding after operations for polypus; but it may be, and is occasionally, required foi the arrestment of spontaneous epistaxis, after all other means, local and general, have failed.

We have found a common gum catheter answer better than a loop of wire, as a means of carrying the string from the posterior nares outwards. It is more flexible and gives less uneasiness.

*Excision of the Tonsils.*

Mr. Liston very properly reprobates the ligature. He recommends excision, and performs it so:—the patient is placed opposite a strong light, the surgeon depresses the tongue with the fore-finger of one hand, and seizes the body of the gland with the vulsellum held in the other. He then introduces the narrow, straight, blunt-pointed knife, which he has held in readiness betwixt his lips, with its edge directed upwards under the gland, and, by a few gentle sawing motions, severs it on a level with the folds of the velum. The instruments, knife, or vulsellum, must be held in the right or left hand, according to the side operated upon. Immediate relief follows this proceeding; it is not by any means so painful as the removal of a tooth, and is not attended with greater loss of blood.

Mr. Liston makes no mention of a very good instrument invented by Dr. Warren, and brought by him to England. We have succeeded in two instances in gradually getting rid of a superabundance of tonsils by (we think) Mr. Cusack's method. It consists in applying a stick of lunar caustic to the tonsils, and pressing it firmly against the gland for a minute or two. A succession of small sloughs is thus produced, which removes the unnecessary portion of gland without pain or any sort of inconvenience. Excision is often no joke in children.

*Foreign Bodies in the Nostrils.*

Foreign substances are often lodged deeply in the nasal fossæ of children, more especially in the fissure betwixt the anterior and posterior cavities, such as seeds, kernels of fruit, glass beads, &c., and they, often enough, are impacted more firmly by awkward and ill-directed efforts to remove them by forceps. The membrane swells and becomes soon tender. The foreign body may absorb moisture and increase in size, and the difficulty of getting rid of it is thus much increased. At any period, by placing a small bent scoop or curette behind the substance, it can be readily enough dislodged; it may slip into the throat, but generally it can be made to appear at the anterior opening. The patient must be firmly secured and a good light chosen; the extraction can then be effected without much pain or trouble; his struggles and screams are disregarded, for the object must be accomplished in the first instance. The difficulties are all increased by delay and repeated futile attempts.

*Foreign Bodies in the Œsophagus.*

Small sharp substances, as pins, needles, fish-bones, are generally fixed in the mucous membrane, over the root of the tongue, by the base of the arches of the velum, and within reach of the finger; they can generally be disentangled and removed by the skilful use of a pair of long and slightly-bent forceps guided by the finger. Foreign matters, of large size, impacted in the narrow part of the canal, can be, if of soft consistence, pushed down by the introduction of a probang, a small bit of sponge, or what is better, a small ovoid and smooth piece of ivory, firmly secured on a whalebone rod. In introducing this instrument, the head and face are so placed, well held back, that the canal is brought pretty much into a straight line. The patient is desired to make an effort to swallow as the ball is passed over the root of the tongue; it is thus easily slid over the epiglottis, and enters the gullet. The pressure must be very gentle on the obstructed part. If the passage has been previously constricted, a smaller instrument of the same kind may be tried, or an œsophagus bougie substituted. Hard an angular bodies must be brought upwards, and for the purpose of seizing these, long, bent forceps, one pair made to open laterally, another from behind forwards, will be required to insure a successful issue to the attempt. The finger is passed as a guide, and the same precautions are taken to avoid interference with the glottis, as in the use of the probang. The cause of the obstruction is felt, grappled with, and seized. The extraction of pieces of bone, or other sharp and irregular bodies, as glass or broken china, must be cautiously gone about; the dread of still further injuring important parts being before the eyes of the surgeon. Flat and smooth substances, coins, &c., may be disentangled readily by a flat, blunt hook, fixed on a piece of whalebone, or by a piece of thick, flexible wire, twisted and bent to a convenient form. A foreign substance has been so fixed in the gullet that it has been considered impossible or unsafe to dislodge it upwards or downwards. Nourishment could not be received into the stomach, and the respiration, it has been thought, might thus be interfered with by pressure on the posterior membranous surface of the air-tube. But the obstacle is almost uniformly lodged behind the cartilages of the larynx, which are pretty incompressible. In these cases an incision has been made into the œsophagus from the side of the neck, on its left side, the foreign body has been felt, and care being taken to steer clear of the vessels and nerves, more especially the inferior thyroid, and the recurrent, the tube has been cut through and extraction accomplished. The necessity for this proceeding can seldom arise.

Here we must pause. There are a thousand excellent hints in this volume, which is an eminently practical manual of operative surgery, and ought to be in the hands of every surgeon. The rapid sale of the first edition shews the estimation in which it is held, and we venture to prophecy an equally rapid sale of this and of future ones. We recommend the book in the most cordial manner to all our surgical readers—and to the old as well as to the young.

☞ The Second Edition of Mr. Liston's "Elements of Surgery" came to hand while this sheet was passing through the press. We shall notice it in our next.

II. A PRACTICAL TREATISE ON THE DISEASES OF THE EYE. By *William Mackenzie*, M.D. Surgeon Oculist in Scotland in ordinary to Her Majesty, Lecturer on the Eye in the University of Glasgow, and one of the Surgeons to the Glasgow Eye Infirmary. To which is prefixed AN ANATOMICAL INTRODUCTION EXPLANATORY OF A HORIZONTAL SECTION OF THE HUMAN EYEBALL. By *Thomas Wharton Jones*, Surgeon. Third edition, octavo, pp. 923. Longman and Co. London.

We find the following advertisement to the present edition of this excellent work:—

The demand for this *Treatise on the Diseases of the Eye* has been much greater than the author had any reason to expect. Since its first appearance, two large editions have been exausted; it has also been reprinted at Boston in America, and a German translation of it has been published at Weimar.

In the present edition, new matter, which would have extended to about 140 pages of the second, has been added; but, by enlarging the page, the work has been kept within its former bounds. The whole has again been carefully revised, and such alterations made as have been suggested by the author's continued experience, or by his perusal of the writings of others. He trusts that it will be found, that, on every occasion, he has endeavoured to treat his fellow-labourer's in the same field with becoming deference and perfect fairness; never appropriating to himself the labours and improvements of others, but acknowledging openly what he has borrowed.

His friend, Mr. T. Wharton Jones, has revised his *Horizontal Section of the Eyeball*, and *Anatomical Introduction*, comprehending in the latter the most recent discoveries in the anatomy of the eye. Mr. Jones has also furnished drawings for a number of new wood-cuts in the body of the work, for which as well as for various practical hints from him and several other medical friends, the author takes this opportuninty of returning thanks.

In its present shape, this must be regarded as the most complete Treatise on the Diseases of the Eye which we possess. Its value is enhanced by the woodcuts, a mode of illustration which must be daily more employed in elementary works. In a future edition, it might be well to add some coloured lithographic representations of some of the diseases of the eye. Were this done the work would be even more valuable than it is—no trivial commendation. We recommend this edition to our readers in the strongest manner.

III. ELEMENTS OF THE THEORY AND PRACTICE OF MEDICINE: DESIGNED FOR THE USE OF STUDENTS AND JUNIOR PRACTITIONERS. By *George Gregory*, M.D. Fellow of the Royal College of Physicians, in London; Physician to the Small-pox and Vaccination Hospital; and Lecturer on the Principles and Practice of Medicine in London. Fifth edition, pp. 750. London, Renshaw, Strand.

Our old friend Dr. Gregory has appeared with *his* New Edition on the scene. We are always happy to meet him, and more particularly on such an occasion. His work has ever been a favourite, from the pleasing character of the style and the perspicuity of the matter. And we venture to say it will now be more a favourite than ever. In his preface, Dr. Gregory informs us:—

The demand for a fifth edition has afforded the author an opportunity of making some alterations in the work, which he hopes will give it additional value. The several diseases of the human body have been thrown into a new arrangement. The distinction between acute and chronic diseases has been abandoned, and a classification has been adopted better suited to the existing state of medical science. The *formulæ medicamentorum* scattered throughout the work have received some additions, and have everywhere been adapted to the nomenclature of the new London Pharmacopœia. Five new chapters have been added—namely, on Cachexia, Hypochondriasis, Cynanche Cellularis, Otitis, and Furuncular Inflammation. The results of the author's more recent experience have been incorporated into the text, and such other information introduced as appeared calculated for elementary instruction. The industry and talents of cotemporary writers, however, it must be observed, are chiefly displayed in matters of minute detail, which, though full of value to the practising physician, are scarcely fitted for a work the scope of which does not extend beyond the fundamental principles of practical medicine.

Dr. Gregory makes some other observations in his Preface, which may claim a passing notice. He remarks for instance that—

Fifty years have elapsed since the death of Dr. Cullen, during which long period a general spirit of improvement has pervaded every branch of medicine, and many important changes have taken place in the opinions entertained regarding the origin of diseases, the modes of detecting them, their seats, and effects. These constitute what may be called the great body of pathology, and it is here that we shall find those improvements which modern medicine may boast. Nor must it be supposed that improvements in pathology are necessarily followed by corresponding changes in the methods of treating disease. These, it has long been observed, have continued nearly the same through every variety of pathological doctrine. It is enough to say that the powers of medicines do not necessarily keep pace with the powers of the human mind in investigating the causes and tracing the relations of diseases.

Of the truth of this remark, at all events in one sense, there can be, we apprehend, no doubt. Our specifics have not increased in number, nor has our confidence augmented in degree. Yet our treatment of diseases has been much improved notwithstanding. In the first place, all that relates to the prevention of disease is much better understood. In the second, the operation of medicines is much more accurately comprehended; witness the use of mercury in inflammatory diseases, as well as in venereal, the exhibition of opium, &c. In the third place we have new medicines of great value, or new preparations of the old. Both in medicine and in surgery, it would be unjust to say that the treatment of disease has not materially improved, though we quite agree with Dr. Gregory that it is in the discrimination of disease that most progress has been made. Speaking of the *Sources of Disease*, Dr. Gregory observes :—

" Great additions have been made since Dr. Cullen's time to our stock of knowledge regarding the sources of disease, both those which are exterior to the frame and those which have their seat within the human body itself. The doctrine of morbid poison, miasma, and contagion, has been much extended; but perhaps there is no branch of pathology which is still in so defective a state. The remarkable differences of opinion entertained on all questions of contagious influence are but little creditable to an age which may boast of such precision in other departments of the science. Two diseases unknown to Dr. Cullen have sprung into notice since his time; namely, Cow-pox and Malignant Cholera. With regard to those sources of disease which exist within the frame itself, we may specify the following as being little, if at all, known to the writers of Cullen's age :—spinal irritation; disease of the brain leading to purulent deposits ; spontaneous inflammation of veins and arteries, and the several varieties of disorganized heart."

It were indeed to be desired that medical philosophers would agree on some first principles in determining the laws of contagious diseases. Unfortunately, there are difficulties attendant on our means of investigating the subject, independently of the difficulties that are inherent in itself.

We are glad to perceive that Dr. Gregory does justice to auscultation and percussion in diseases of the chest. His testimony in their favour is the more to be prized, because he was disposed at first to question their utility.

" Much has been done, especially during the last twenty years, towards the more accurate detection of disease. Some progress has been made in the minute diagnosis of diseases of the brain, but it is in the disorders of the thoracic viscera where this great improvement is most manifest. The several diseases of the heart and lungs are now distinguished with an accuracy which would have surprised the most experienced physicians of former times, though one of them (Baglivi), possessed of more than ordinary sagacity, foresaw its possibility. For the original discovery of the healthy murmur of respiration, and of the several modifications which it undergoes during disease of the chest, we are indebted to the acuteness and patient observation of Laennec. To the same enlightened pathologist we owe much of our knowledge regarding the sounds which issue from the heart during disease. This branch of the great subject of medical acoustics is still pursued with diligence, and gives the promise of ultimately attaining to great perfection."

We were always of opinion, and still are so, that auscultation of the heart will ultimately furnish more satisfactory data than auscultation of the lungs. The observations we shall now quote (and we conclude by doing so) on pathology and on morbid anatomy exactly represent the sentiments we have more than once expressed.

" This improvement in modern pathology may be considered as commencing with Dr. Baillie, who succeeded in directing the attention of physicians to the study of morbid anatomy more effectually than had been done by Morgagni, his laborious but diffuse predecessor in the same department of science. It must be acknowledged, at the same time, that the example thus set has been diligently followed in France; and to the zeal of the French pathologists, and the abundant opportunities afforded by the French Hospitals, we are chiefly indebted for the increased amount and accuracy of our information regarding the changes of structure effected by disease.

To such an extent has this study been lately followed, that the term pathology has been, by some, considered as identical with morbid anatomy, and the attention of students has been fixed upon the dissecting-room, as if the elements of pathology could there only be properly studied. This, however, (as a most able writer and acute pathologist has pointed out,) is a manifest error in science. The alterations in structure produced by disease are only one of the elements of our reasoning on the nature of diseased actions. Many other considerations are equally necessary to their elucidation, such as the external causes of disease, the nature of the leading symptoms, their consequences, local and general, and the effects of remedies. These severally furnish the proper elements of that inductive reasoning by which we determine the laws of the animal economy during the presence of disease.

It is also, as the same author observes, a great practical error to fix the attention of students too exclusively upon the *effects* of disease, because in many instances their characters during life are not perceived until the latest and least remediable stage of the disease. The most important part of the history of a disease is that group or assemblage of symptoms by which its nature and seat may be known before any decided lesion of structure has taken place; for this is the season of successful practice, and the mind of the physician should never be diverted from those primary objects of his pursuit,—the power of remedies, and their application during life, to control diseased actions, or to relieve the urgency of particular symptoms."

We have skimmed over the volume, and it seems to us amply to bear out the promise of its author. It is a work admirably calculated for students, with whom we are sure it will be an especial favourite.

Elements of Natural Philosophy; being an Experimental Introduction to the Study of the Physical Sciences. By *Golding Bird*, M.D. F.L.S. F.G.S. &c. London, John Churchill, 1839.

Dr. Bird is a young physician of very high promise. We know of few in the profession more likely to adorn it by either intelligence or industry. Such a work as the present may come naturally from his hands, and deserves to be, and will be well received.

Dr. Bird informs us in his preface that—

"The following manual is chiefly intended as a text-book for the student whilst attending lectures on physics, or as preparatory to his entering upon the study of larger, and more elaborate works. With this view it has been written; and as the great difficulty experienced in executing this task has arisen from the necessity of knowing, not what to insert, but what to omit, whenever a doubt has arisen on this point, it has been determined by a reference to the amount of knowledge required of the student by the different English and Scottish medical boards."

He adds:—

"I have been greatly indebted to several writers in the French and German languages, for many suggestions and illustrations, of which I have never hesitated to avail myself whenever they appeared to divest any subject of obscurity, or to add to its interest. To the 'Précis de Physique' of Biot, the 'Eléments de Physique' of Pouillet, the 'Traité de Physique' of Hauy, the 'Positions de Physique of Quetelet, and the 'Grundriss der Experimental-Physik' of Kastner, I have been peculiarly indebted for several illustrations, some of which have not, I believe, previously appeared in an English dress.

Having thus explained the object and unpretending character of this volume, I trust enough has been said to blunt the edge of criticism, should such, per chance, be levelled against it. The critic himself, I would beg to remind of the celebrated observation of Horace—

'Sunt delicta quibus non ignovisse velimus,
Nam neque chorda sonum reddit quam vult manus et mens;
Nec semper feriet quodcunque minabitur arcus.'

Those readers who desire further information on the subjects treated of in this work, and have not the assistance derived from attendance on lectures, may, if only a popular acquaintance with them be required, refer to the very elegant, although as yet unfinished, 'Elements of Physics' of Dr. Arnott, or to Sir David Brewster's edition of 'Euler's Letters to a German Princess.' Those who require a more profound acquaintance with these important subjects, should consult the books referred to in the body of this volume, as well as to the treatises published by the Society for the Diffusion of Useful Knowledge. The series of Essays written by the professors of King's College, London, now in the course of publication will also furnish most valuable comments on this, and other elementary works."

The work consists of twenty-five chapters, and a preliminary discourse. We believe that the best way of giving an idea in the smallest space of its character will be to present the titles of the chapters.

The *first* is on the General Properties of Atoms and Masses of Matter.—2d, Attractive Forces exerted between Masses.—3rd, Bodies in Motion, or General Dynamics.—4th, Effects of Gravitation.—5th, Theoretical Action of the Simple Machines.—6th, Fluids at Rest, or Hydrostatics.—7th, Gases at rest, Aerostatics, or Pneumostatics.—8th, Fluids in Motion, or Hydro and Pneumo-dynamics.—9th, Sonorous Vibrations of Ponderable Bodies, or Acoustics.—10th, Magnetism.—11th, Primary Phenomena of Ordinary Electricity.—12th, Consequences of Electrical Induction.—13th, Consequences of Electric Induction.—14th, Phenomena of Atmospheric Electricity.—15th, Voltaic Electricity.—16th, Electro-Dynamics.—17th, Electro-dynamic Induction.—18th, Thermo-Electricity.—19th, Organic-electricity.—20th, General Properties and Catoptric Phenomena, of Unpolarized Light.—21st, Dioptric Phenomena of Unpolarized Light.—22nd, Chromatic Phenomena of Unpolarized Light.—23rd, Phenomena of Double Refraction, and Rectilinear Polarization.—24th, Chromatic Phenomena of Polarized Light.—25th, Optical Apparatus, and the Eye considered as an Optical Instrument.

We commend the book to the medical public. They who would wish to acquire or brush up an acquaintance with Natural Philosophy will spend a few shillings excellently well on this little volume.

